# Mediation of Polygenic Asthma Risk Through Gene Expression

**DOI:** 10.1111/all.70101

**Published:** 2025-12-06

**Authors:** Rakesh Natarajan, Brooke Szczesny, Kanika Kanchan, Erika Esquinca, Meher Preethi Boorgula, Sameer Chavan, Monica Campbell, Wendy Lorizio, Ayo P. Doumatey, Alvaro A. Cruz, Harold Watson, Edward T. Naureckas, B. Louise Giles, Ganiyu Arinola, Olumide Sogaolu, Adegoke G. Falade, Nadia N. Hansel, Christopher O. Olopade, Charles N. Rotimi, R. Clive Landis, Camila A. Figueiredo, Eimear E. Kenny, Ingo Ruczinski, Andrew H. Liu, Carole Ober, Margaret A. Taub, Randi K. Johnson, Kathleen C. Barnes, Genevieve L. Wojcik, Rasika A. Mathias

**Affiliations:** ^1^ Genomics and Precision Health Section, Laboratory of Allergic Diseases, National Institute of Allergy and Infectious Diseases National Institutes of Health Bethesda Maryland USA; ^2^ Department of Biomedical Informatics University of Colorado Anschutz Medical Campus Aurora Colorado USA; ^3^ Department of Medicine University of Colorado Denver, Anschutz Medical Campus Aurora Colorado USA; ^4^ Department of Medicine Johns Hopkins University Baltimore Maryland USA; ^5^ Center for Research on Genomics and Global Health, National Human Genome Research Institute National Institutes of Health Bethesda Maryland USA; ^6^ Fundacao ProAR and Federal University of Bahia Salvador Bahia Brazil; ^7^ Faculty of Medical Sciences, The University of the West Indies, Queen Elizabeth Hospital, St. Michael Bridgetown Barbados; ^8^ Department of Medicine University of Chicago Chicago Illinois USA; ^9^ Department of Pediatrics University of Chicago Chicago Illinois USA; ^10^ Department of Immunology, College of Medicine University of Ibadan Ibadan Nigeria; ^11^ Department of Medicine, College of Medicine University of Ibadan Ibadan Nigeria; ^12^ Department of Pediatrics University of Ibadan Ibadan Nigeria; ^13^ University College Hospital Ibadan Nigeria; ^14^ Edmund Cohen Laboratory for Vascular Research, George Alleyne Chronic Disease Research Centre, Caribbean Institute for Health Research The University of the West Indies, Cave Hill Campus Wanstead Barbados; ^15^ Instituto de Ciências de Saúde Universidade Federal da Bahia Salvador Brazil; ^16^ Program for Control of Asthma in Bahia (ProAR) Salvador Brazil; ^17^ Center for Genomic Health Icahn School of Medicine at Mount Sinai New York New York USA; ^18^ Department of Biostatistics Johns Hopkins Bloomberg School of Public Health Baltimore Maryland USA; ^19^ Department of Pediatrics Childrens Hospital Colorado and University of Colorado Denver, Anschutz Medical Campus Aurora Colorado USA; ^20^ Department of Human Genetics University of Chicago Chicago Illinois USA; ^21^ Department of Epidemiology Colorado School of Public Health Aurora Colorado USA; ^22^ Department of Epidemiology Bloomberg School of Public Health, Johns Hopkins University Baltimore Maryland USA

**Keywords:** African ancestries, asthma, eosinophils, gene expression, GWAS, IgE, medication response, polygenic risk scores, T2 inflammation, wound healing

## Abstract

**Background:**

Existing asthma polygenic risk scores (PRSs) have minimal validation in African‐ancestry populations, leaving gaps in our understanding of the wide applicability of PRSs. To widen our understanding of the applicability of asthma PRSs, we apply published PRSs in African‐ancestry individuals and quantify the extent to which the PRS‐asthma relationship is mediated by clinical biomarkers and gene‐expression signatures of asthma.

**Methods:**

We applied 22 PRSs from the PGS Catalog in 673 individuals from the Consortium on Asthma among African‐Ancestry Populations in the Americas (CAAPA) and calculated the percent of the PRS‐asthma relationship that is statistically mediated by clinical and nasal epithelium transcriptomic biomarkers of asthma. Asthma case/control status was defined as ever/never having a doctor's diagnosis of disease. For gene expression mediation analysis, we limited the cases to those with current disease.

**Results:**

The PRS (PGS001782) created by the Global Biobank Meta‐analysis Initiative (*N* = 32,658 individuals of African ancestry) performed the best (ΔAUC = 0.104, AUC = 0.657) adjusted for age, sex, study site, and the first two genetic principal components (PC1‐2). The PRS's effect on asthma was mediated by total IgE (tIgE) (38.8%, *p*.adj < 0.0002), multi‐allergen ImmunoCAP phadiatop specific IgE (sIgE) (38.7%, *p*.adj < 0.0002), and eosinophils (7.3%, *p*.adj = 0.004). Mediation was observed for gene expression modules related to T2 inflammation (21.9%, *p*.adj < 0.0024), wound healing (11.9%, *p*.adj = 0.008), and medication response (6.8%, *p*.adj = 0.049).

**Conclusion:**

We found the best PRS to be the one derived using the largest sample size and including African‐ancestry individuals. Mediation supports the well‐documented biology of T2 inflammation in asthma as well as pathophysiological components of asthma like wound healing and medication response.

## Graphical abstract

We apply published PRSs in African‐ancestry individuals and quantify the extent to which the PRS‐asthma relationship is mediated by clinical biomarkers and gene‐expression signatures of asthma. We found the best PRS to be the one derived using the largest sample size and including African‐ancestry individuals. Mediation supports the well‐documented biology of T2 inflammation in asthma as well as pathophysiology components of asthma like airway wound healing and asthma medication response. △AUC, change in the area under the ROC curve (AUC); CAAPA, Consortium on Asthma among African‐Ancestry Populations in the Americas; GWAS, genome‐wide association studies; IgE, immunoglobulin E; PRS, polygenic risk score; T2, type 2.

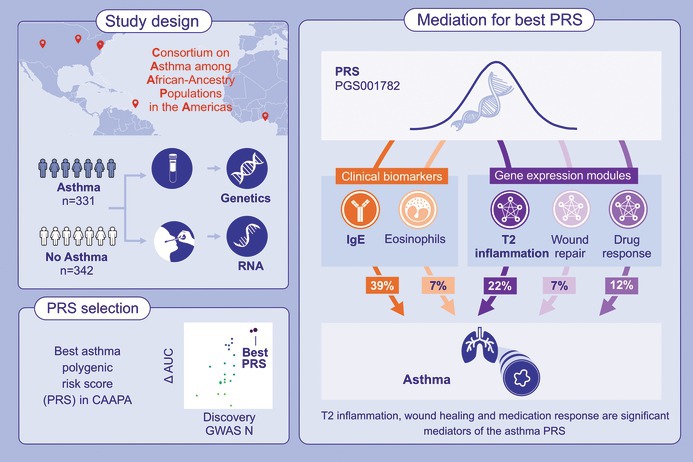

**Abbreviations**
1kG: 1000 Genomes Project
AMR: indigenous American
AUC: area under the receiver operating characteristics curve
BH: Benjamini‐Hochberg
CAAPA: Consortium on Asthma among African‐ancestry Populations in the Americas
CEU: Centre d'Etude du Polymorphism Humain Northern European samples from Utah
DEG: differentially expressed gene
GBMI: Global Biobank Meta‐analysis Initiative
Geo. Mean: geometric mean
GWAS: genome‐wide association studies
PCs: principal components
PGS: Polygenic Scores
PRS: Polygenic Risk Scores
QC: quality control
RIN: RNA integrity number
sIgE: multi‐allergen specific serum Immunoglobulin E
T2: type 2
TAGC: Trans‐National Asthma Genetic Consortium
tIgE: total serum Immunoglobulin E
WGCNA: weighted gene co‐expression network analysis
YRI: Yoruba in Ibadan, Nigeria

## 1 Introduction

Asthma is a common, complex, and chronic disease that is characterized by inflammation of the airways, airway hyperresponsiveness, and bronchospasms. The etiology of asthma involves a complex interplay between genetic and nongenetic risk factors, with heritability between 40% and 80% [1, 2]. Numerous common variants associated with asthma risk have been identified through genome‐wide association studies (GWAS), with estimates of the SNP‐based heritability of asthma ranging up to 33% [3]. GWAS has enabled the development of polygenic risk scores (PRSs), weighted sums of common variants that are designed to predict a phenotype with potential clinical applications [4, 5, 6].

Performance of PRS can vary across populations due to differences in genetic similarity, environmental risk factors, and disease (e.g., endotype distributions, diagnosis criteria, etc.) between validation populations [7, 8]. Concerns about the variability of PRS performance between populations are especially concerning for asthma due to the condition's multiple axes of clinical variation [3, 7, 8, 9].

The lack of ancestry variability in discovery GWAS datasets has been firmly established; specifically, no asthma PRS in the PGS Catalog was trained off > 6% African‐ancestry samples. This issue also extends to the validation of PRSs; the PGS Catalog includes no validation datasets where > 25% samples were reported as African ancestry, despite African ancestries being among the most genetically diverse populations in the world [10]. Among the validation samples cited in the PGS Catalog, the African‐ancestry cohorts used to evaluate asthma PRSs were exclusively from the UK Biobank, representing a small subset of the genetic variation found within the African diaspora and suffering from known participation biases [11, 12, 13]. This raises notable concerns about whether existing performance metrics for asthma PRSs are generalizable beyond the UK Biobank and limits our understanding of the genetic basis of asthma when viewed in light of existing health disparities [14, 15, 16, 17, 18].

The Consortium on Asthma among African‐ancestry Populations in the Americas (CAAPA) seeks to discover genes and mechanisms conferring risk to asthma in populations of African ancestries, utilizing multi‐omic data. Recently, we used a multi‐omics approach in nasal epithelium to confirm T2 mechanisms in asthma risk, and also identified novel wound healing and medication response signatures, providing new information about the biological mechanisms underlying asthma [19]. It remains unclear if these novel signatures of biological mechanisms found in CAAPA are captured by the existing asthma PRSs.

Here, we applied existing PRSs to African‐ancestry samples from CAAPA not only to evaluate the generalizability of asthma PRSs but also to follow up on the novel asthma mechanisms previously uncovered via transcriptomics approaches. Our goal with integrating PRS and transcriptomic signatures through mediation analyses is to examine asthma PRSs beyond the traditional framework of T2 versus non‐T2 asthma, instead looking at the PRSs in relation to the three transcriptomic axes of dysregulation.

## 2 Methods

### 2.1 Study Subjects and Case Definitions

Study subjects were from CAAPA, an asthma case–control study (*N* = 673) comprised of adult (ages 18–89; *N* = 471) and pediatric (ages 8–17; *N* = 202) individuals who self‐identified as African, African American, African Caribbean, African Brazilian, or African‐Other from four US sites (Denver, Baltimore, Washington DC, Chicago) and three non‐US sites (Barbados, Nigeria, and Salvador in Brazil). Detailed information about study recruitment, inclusion and exclusion criteria, sample collection, data collection, quality control, RNA sequencing preprocessing, ancestry deconvolution (ADMIXTURE), and principal components analysis of CAAPA were previously published [19]. The primary phenotype definition utilized was ever asthma, which was defined based on a self‐report of physician‐confirmed ever asthma (an affirmative response to both of the following questions: “Have you ever had asthma?” and “Was it confirmed by a doctor?”). The Composite Asthma Severity Index (CASI) takes into consideration medication use and the corresponding treatment level in determining asthma severity [20]. The CASI questionnaire was used to subset asthma cases to those with current asthma. The current asthma case definition was defined by subsetting ever asthma cases to those with a CASI score ≥ 1. The controls for both the ever asthma and current asthma analyses were identified as individuals who responded “no” to the question: “Have you ever had asthma?” The current asthma case definition was used for the transcriptomic mediation analyses since the nasal epithelium transcriptome would largely capture the effects of current disease, while the ever asthma case definition was used for all other analyses. Atopy was defined as total serum IgE (tIgE) > 100 kU/L defined based on Wong et al. [21] and/or multi‐allergen ImmunoCAP phadiatop specific serum IgE (sIgE) ≥ 0.36 PAU/L per assay protocol recommendations.

All samples used for this study were obtained following written informed consent from participants. The University of Colorado (IRB#: 17‐1807), Johns Hopkins University (IRB00179053), University of Chicago (IRB18‐0466‐CR001), National Institutes of Health (IRB#: P184385), University of West Indies (IRB#: 190604‐A), University of Bahia (IRB#: 3.302.487), and University of Ibadan Institutional Review Boards approved the conduct of this study (IRB18‐0840).

### 2.2 Gene Expression Modules

We utilized the weighted gene co‐expression network analysis (WGCNA) gene expression modules that were previously generated within CAAPA by Szczesny et al. [19] using nasal epithelium gene expression on the CAAPA samples. Nasal epithelium brush samples were examined on a slide smear and only samples with > 80% columnar cells representing epithelium were moved forward to RNASeq. We utilize the nasal epithelium as a proxy for the more biologically relevant lung tissue. Poole et al. have demonstrated that nasal airway gene expression profiles largely recapitulate expression profiles in the lung airways [22]. In a meta‐analysis approach Tsai et al. [23] found the magnitude of differential expression was highly similar in bronchial and nasal airway epithelia, and we have shown in our prior CAAPA work that our identified genes are robust to replication evidence from this work by Tsai et al., supporting our use of nasal epithelium [19].

Of the 673 CAAPA samples with genetic data used in the PRS here, 536 had gene expression and existing modules available from Szczesny et al. [19] Here, we tested for association with each of these pre‐computed 24 modules in the CAAPA samples with the Global Biobank Meta‐analysis Initiative (GBMI) asthma PRS, adjusting for age, sex, library preparation batch, site, RNA integrity number, GC content, and genetic PC1‐2. *p*‐values were corrected for multiple testing via the Benjamini‐Hochberg procedure. Modules are described by the most significantly differential gene, the WGCNA hub gene, and the STRING hub gene for comprehensiveness.

### 2.3 Quantitative Trait Measurement

Whole blood collected in BD Vacutainer EDTA tubes was used to perform a complete blood count (CBC) with differentials at each study site's clinical laboratory. The Johns Hopkins University School of Medicine Reference Laboratory for Dermatology, Allergy and Clinical Immunology (DACI) measured tIgE and multi‐allergen ImmunoCAP phadiatop sIgE using serum samples. We assigned phadiatop sIgE and tIgE measurements that fell below the detection limit of the assay (phadiatop sIgE: < 0.1 PAU/L, tIgE: < 2.00 kU/L) to half of the detection limit.

### 2.4 Genotyping, Imputation, and Quality Control (QC)

Genotyping was performed on DNA extracted from blood clots in CPT tubes using Illumina's Multi‐Ethnic Global BeadChip (MEGA) array. Sample and variant QC for the CAAPA genotypes has been previously described [19]. Samples were excluded due to missingness > 3%, sex mismatch, excess heterozygosity, and unexpected relatedness; SNPs were excluded due to missingness > 5%, < 1% minor allele frequency (MAF), and Hardy Weinberg *p* < 1 × 10^−6^. Genotypes were phased using Eagle 2.4 and imputation was conducted on the TopMed Imputation Server using Minimac 4 (version 1.0.2) and the TopMed‐r2 (version 1.0.0) multi‐ancestry imputation panel [24, 25, 26, 27]. Poorly imputed variants (*R* ^2^ < 0.7) were excluded from PRS calculation.

### 2.5 PRS Calculation

PRSs were calculated using only autosomal variants, excluding multi‐allelic, ambiguous, and duplicated variants. The allele frequency of variants in the gnomAD 1000 Genomes + Human Genome Diversity Project reference was used for the dosages of any variants missing within CAAPA. We calculated all asthma (trait: MONDO_0004979) PRSs deposited in the PGS Catalog on November 13, 2023, with a variant match rate > 75% in the CAAPA samples. All polygenic scores were converted into *Z*‐scores for subsequent analyses. PRSs were calculated using the Polygenic Score Catalog Calculator (PGSC_Calc), version 2.0.0‐alpha2 [11, 28]. The discovery GWAS used in the PRS did not have any overlap with CAAPA data in this paper.

### 2.6 PRS Performance Evaluation

For each PRS, an area under the receiver operating characteristic curve (AUC) was calculated based on a logistic regression of ever asthma within CAAPA adjusting for genetic PC1‐2 and non‐genetic covariates (age, sex, study site). The AUC was calculated for both a base model that only included covariates and an expanded model that included covariates and the PRS. An incremental Nagelkerke's *R* ^2^ was calculated based on a logistic regression of ever asthma on the PRS, age, sex, study site, and genetic PC1‐2. PRS performance was evaluated using R 4.3.0 and the following R libraries: fmsb 0.7.6 and pROC 1.18.5 [29, 30, 31].

### 2.7 PRS Mediation Analysis

The percentage of the PRS‐asthma relationship that was mediated by several variables was determined using the mediation method proposed by MacKinnon et al. [32]; this method consists of three regressions that determine the PRS‐Mediator, PRS‐Asthma, and PRS‐Asthma (adjusted for mediator) associations. All regressions were adjusted for age, sex, study site, and genetic PC1‐2. *p*‐values and 95% confidence intervals for the percent mediation were obtained from 10,000 bootstrap samples. *p*‐values were corrected for multiple testing via the Benjamini‐Hochberg procedure. Mediation analyses were performed using the PRS that performed the best within CAAPA (PGS001782).

We considered the 24 gene expression modules from Szczesny et al. [19] and relevant quantitative biomarkers (eosinophils, neutrophils, tIgE, and phadiatop sIgE) as potential mediators. Biomarker mediation analyses were performed using subsets of study participants with non‐missing quantitative biomarker values (*N* = 639–670). Gene expression mediation analyses were performed using the current asthma subset (*N* = 536).

## 3 Results

### 3.1 Study Participants

The overall CAAPA study population used to evaluate PRS performance consisted of 331 ever asthma cases and 342 never asthma controls. Both the cases and controls had comparable distributions of sex, age, and study site (Table 1). Additionally, ADMIXTURE estimates indicated that both groups had comparable levels of genetic similarity to reference panel samples. Cases were more likely to be atopic (80.7% vs. 44.4%, *p* < 0.001) and have higher eosinophil counts (Geo. Mean: 160.63 vs. 105.63 cells/mm^3^, *p* < 0.001), tIgE counts (Geo. Mean: 108.81 vs. 28.03 kU/L, *p* < 0.001), and phadiatop sIgE (Geo. Mean: 3.16 vs. 0.30 PAU/L, *p* < 0.001) counts, compared to controls. Clinical characteristics for the case/control subset used for the transcriptomic mediation analysis are shown in Table S1 and showed similar patterns to the full dataset for all clinical characteristics.

**TABLE 1:** Clinical characteristics of CAAPA study participants. *p*‐values are presented for the quantitative clinical traits used in the mediation analysis, derived using a 2‐sample *t*‐test.

Clinical characteristic	Ever asthma case (*N* = 331)	Never asthma control (*N* = 342)	Overall (*N* = 673)	*p*
Age (years)				
Mean (SD)	32.24 (17.77)	31.71 (16.50)	31.97 (17.12)	
Sex, *N* (%)				
Female	198 (59.8%)	206 (60.2%)	404 (60.0%)	
Study site, *N* (%)				
Brazil	47 (14.2%)	51 (14.9%)	98 (14.6%)	
Denver	53 (16.0%)	52 (15.2%)	105 (15.6%)	
Chicago	51 (15.4%)	48 (14.0%)	99 (14.7%)	
Baltimore	49 (14.8%)	43 (12.6%)	92 (13.7%)	
Washington DC	31 (9.4%)	51 (14.9%)	82 (12.2%)	
Barbados	50 (15.1%)	50 (14.6%)	100 (14.9%)	
Nigeria	50 (15.1%)	47 (13.7%)	97 (14.4%)	
Percent similarity to 1kG‐YRI				
Mean (SD)	0.80 (0.19)	0.79 (0.20)	0.79 (0.20)	
Percent similarity to non‐admixed Mao, et al. AMR samples				
Mean (SD)	0.02 (0.04)	0.02 (0.04)	0.02 (0.04)	
BMI (kg/m^2^)				
Mean (SD)	29.37 (14.45)	27.71 (24.83)	28.52 (20.78)	N.S.
Missing, *N* (%)	23 (3.4%)	10 (2.9%)	13 (3.9%)	
Percent similarity to 1kG‐CEU				
Mean (SD)	0.18 (0.17)	0.19 (0.18)	0.19 (0.18)	
BMI (kg/m^2^)				
Mean (SD)	29.37 (14.45)	27.71 (24.83)	28.52 (20.78)	N.S.
Missing, *N* (%)	23 (3.4%)	10 (2.9%)	13 (3.9%)	
Atopy status, *N* (%)				
Atopic	267 (80.7%)	152 (44.4%)	419 (62.3%)	< 0.001
Missing, *N* (%)	2 (0.6%)	1 (0.3%)	3 (0.4%)	
Eosinophils (cells/mm^3^, offset of +1)				
Geo. mean (Geo. CV)	160.63 (169.9)	105.63 (153.5)	129.58 (166.1)	< 0.001
Missing	16 (4.8%)	11 (3.2%)	27 (4.0%)	
Neutrophils (cells/mm^3^, offset of +1)				
Geo. mean (Geo. CV)	2743.33 (59.1)	2609.79 (69.5)	2673.12 (64.6)	0.0859
Missing, *N* (%)	24 (7.3%)	10 (2.9%)	34 (5.1%)	
tIgE (kU/L)				
Geo. mean (Geo. CV)	108.01 (336.6)	28.03 (300.1)	54.37 (405.2)	< 0.001
Missing, *N* (%)	2 (0.6%)	1 (0.3%)	3 (0.4%)	
Phadiatop sIgE (PAU/L)				
Geo. mean (Geo. CV)	3.16 (1580.3)	0.30 (1283.4)	0.95 (2836.9)	< 0.001
Missing, *N* (%)	3 (0.9%)	1 (0.3%)	4 (0.6%)	

Abbreviation: Geo., geometric.

### 3.2 PRS Calculation and Performance Evaluation

Of the 30 PRSs published in the PGS Catalog at the time of this analysis (November 23, 2023), six scores were excluded due to a variant match rate < 75% in CAAPA. Of the remaining 24 calculated scores, between 77.2% and 100% (median: 90.5%) of variants in the PRS score files were found in CAAPA and passed QC; 20 scores had a variant match rate > 85% (Table S2).

**FIGURE 1:**
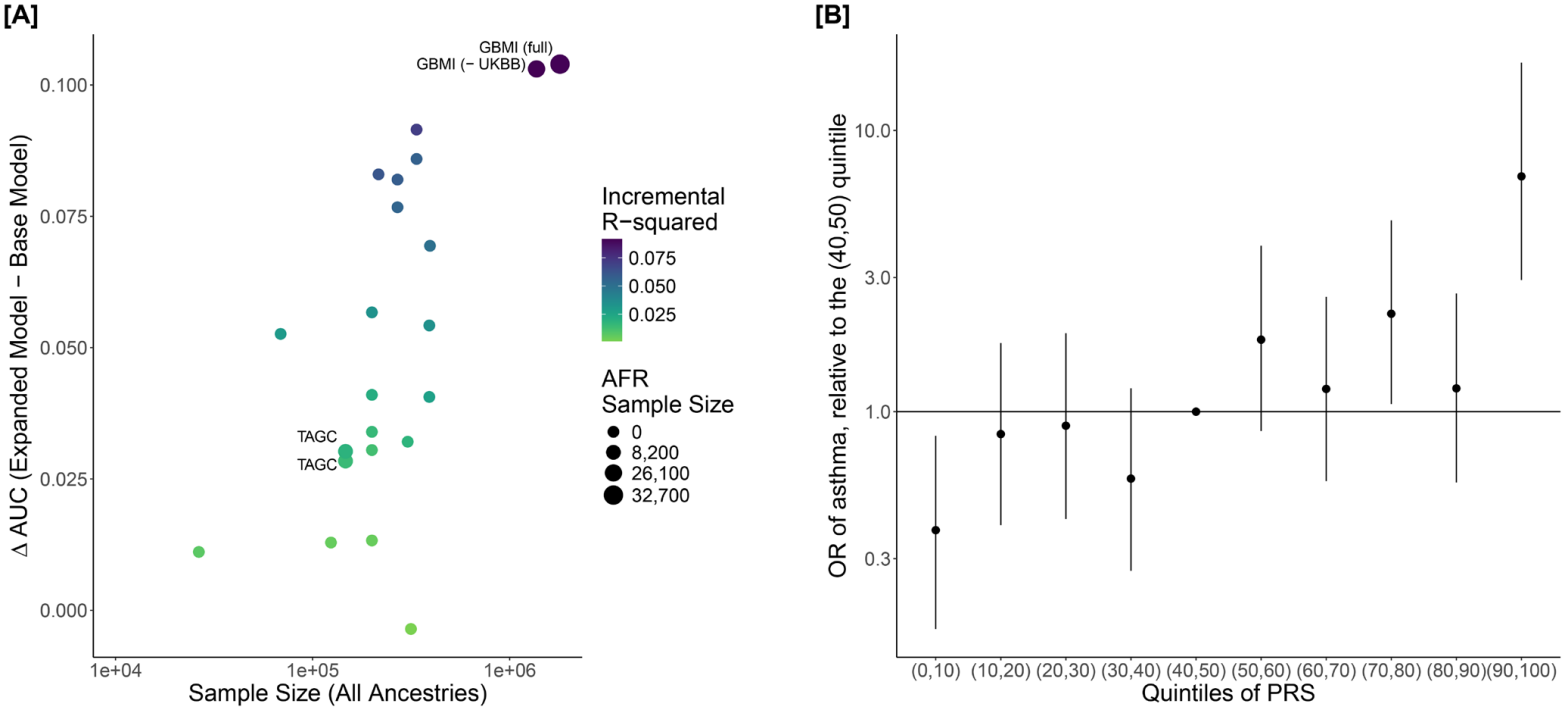
(A, B) PRS performance within CAAPA. Panel A shows the discriminative performance of PRSs within CAAPA, contrasting the change in the area under the ROC curve [ΔAUC = AUC_Expanded Model_−AUC_Base Model_] within CAAPA (*y*‐axis) against the sample size of the PRS's discovery GWAS (*x*‐axis). Scores that utilized any (*n* > 0) AFR samples were labeled with the study name. The size of the points is based on the number of AFR samples included in the PRS training/development, rounded to the nearest 100. Scores are colored based on the incremental Nagelkerke's *R* ^2^. The base model consists of age, sex, study site, 2 genetic PCs, while the expanded model consists of the base model + PRS. Panel B shows the odds ratio for asthma comparing deciles of the GBMI full‐cohort PRS (PGS001782), using the (40, 50) percentile as a reference, broadly demonstrating that the odds ratio of asthma is higher among higher deciles of the PRS. Odds ratios are adjusted for age, sex, study site, and genetic PC1‐2.

As shown in Figure 1A, Figure S1 and Table S3, there was a positive correlation between the PRS development sample size and their performance as assessed by ΔAUC, odds ratios, and incremental Nagelkerke's *R* ^2^ within CAAPA (*r*_Spearman_ = 0.644, *p* = 0.0012). The PRS (PGS001782) with the highest AUC within CAAPA was from the GBMI [8]; the AUC of this expanded model including the PRS and covariates was equal to 0.657 (95% CI: 0.616–0.698), which is 0.104 greater than the base model (AUC = 0.553, 95% CI: 0.509–0.596) that only included the covariates. This PRS had not only the largest overall development sample size but included the most African‐ancestry samples (*N* = 1,800,785; *N*_AFR_ = 32,658). The odds ratio of asthma associated with a standard deviation (SD) increase in the PRS within CAAPA was 1.797 (*p* = 4.18 × 10^−11^), adjusted for age, sex, study site, and genetic PC1‐2. Individuals within a higher decile of the GBMI asthma PRS generally had a larger odds ratio for asthma (Figure 1B), relative to the (40th, 50th) decile; the (90th, 100th) decile has the largest risk with an OR of 6.87 (95% CI: 2.94–17.42). Because this PRS had the best performance in CAAPA, it was used in all downstream mediation analyses.

### 3.3 Quantitative Trait Mediation

By comparing models with and without potential mediators (eosinophils, neutrophils, tIgE, phadiatop sIgE), we assessed whether quantitative traits mediated the PRS‐asthma relationship. As shown in Table 2, eosinophils, tIgE, and phadiatop sIgE were all statistically significant (*p*.adj < 0.05) mediators of the PRS‐Asthma relationship; neutrophils were not a statistically significant mediator. Serum tIgE and phadiatop sIgE were the strongest mediators, mediating 38.8% (95% CI: 25.8%–56.8%) and 38.7% (95% CI: 26.5%–54.6%) of the PRS‐Asthma relationship, respectively. Eosinophils mediated 7% (95% CI: 2.0%–15.7%) of this relationship. This parallels the observation that as traits themselves, tIgE, phadiatop sIgE, and eosinophils were the most significantly different by asthma, while neutrophils had no statistically significant difference (*p* = 0.11) (Table S1).

**TABLE 2:** The amount of mediation done by each of the quantitative traits.

Mediators/Quantitative traits	Percent mediation (95% CI)	*p*	*p*.adj
tIgE*	0.388 (0.258, 0.568)	< 0.0001	< 0.0002
Phadiatop sIgE*	0.387 (0.265, 0.546)	< 0.0001	< 0.0002
Eosinophils*	0.073 (0.02, 0.157)	0.003	0.004
Neutrophils	0.007 (−0.012, 0.032)	0.5	0.5

*Note:* Mediators that are statistically significant (*p*.adj < 0.05) are indicated with a * in the first column. An offset of +1 was added to all eosinophil and neutrophil measurements. All potential mediators were log‐transformed and converted to *z*‐scores.

Abbreviation: *p*.adj, Benjamini‐Hochberg corrected *p*‐values.

### 3.4 Gene Expression Module Analyses

**FIGURE 2:**
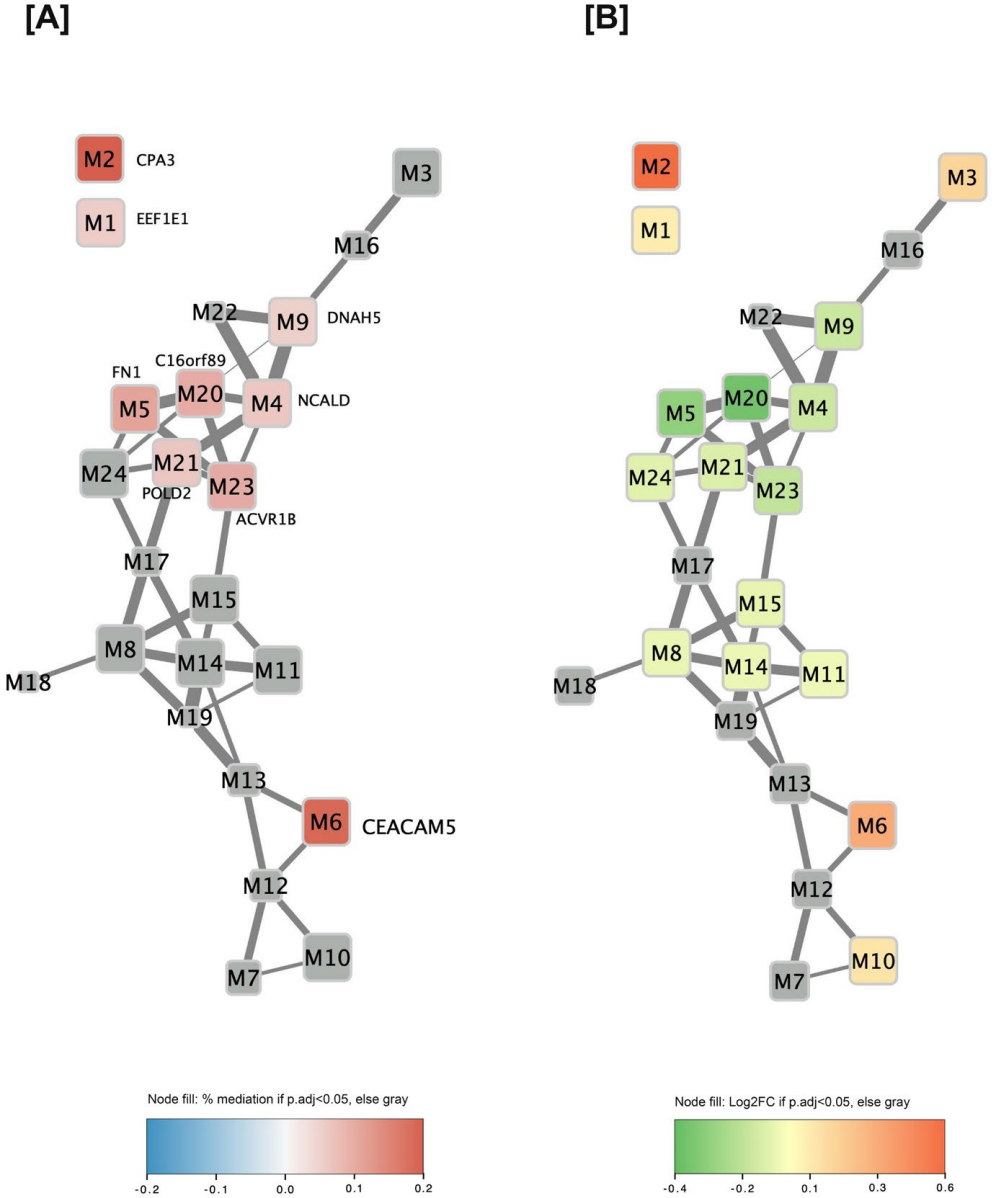
(A, B) CAAPA module connectivity. Each node represents a WGCNA module and each edge represents a significant positive Pearson's pairwise correlation between module expression (*r* > 0.5). Hub genes for modules that were statistically significant mediators are labeled in Panel A. Modules that are not statistically significant are shown in gray for each respective panel. Node size is based on the BH‐corrected *p*‐value and node color is based on the effect size (only for statistically significant modules). Panel A depicts each module's percent mediation, while Panel B shows the logFC for the association between each module and the asthma phenotype, modified from Szczesny et al. [23].

We then further explored the role of specific biological mechanisms mediating the relationship between PRS and asthma for all 24 gene expression modules. Whereas 16 modules were previously shown to be associated (*p*.adj < 0.05) with asthma (Figure 2B and Table S4), only six gene expression modules (M2, M5, M6, M20, M21, and M23) were significantly (*p*.adj < 0.05) associated with the GBMI asthma PRS (Table S4). In the mediation analysis, nine modules (M1, M2, M4, M5, M6, M9, M20, M21, and M23) were statistically significant (*p*.adj < 0.05) mediators of the PRS‐asthma relationship (Figure 2A). Modules 2 (hub gene: *CPA3*) and 6 (hub gene: *CEACAM5*), which relate to T2 inflammation, were the strongest mediators (Figure 2A), mediating 21.99% (95% CI: 12.1%–35.9%) and 20.66% (95% CI: 10.4%–33.8%) of the PRS's effect, respectively. Modules 4 (hub gene: *NCALD*; 6.8%, *p*.adj = 0.049) and 5 (hub gene: *FN1*; 11.9%, *p*.adj = 0.008), which are related to impaired drug response and wound healing, respectively, were among the statistically significant mediators of the PRS‐asthma relationship.

## 4 Discussion

In this paper, we examined the performance of existing asthma PRSs in a dataset recruited from populations of African ancestries. We identified the GBMI PRS (PGS001782) to be the best performing score (AUC = 0.657). This GBMI PRS achieved similar performance in non‐African populations (e.g., AUC = 0.659 in the CanPath European ancestry cohort [33]). While these AUCs are good, the performance still falls short of existing non‐genetic clinical risk prediction models, posing a notable barrier for the clinical implementation of asthma PRSs [34]. The performance of the GBMI PRS in CAAPA surpassing other PRSs is unsurprising as it had the largest training sample size; PRS power calculations show that the training set sample size is positively associated with the area under the ROC curve, explaining the positive, statistically significant correlation between training sample size and the AUC observed within CAAPA [35, 36]. Although we identified that the GBMI PRS performed best within CAAPA, we caution against generalizing these results to all African‐ancestry populations because populations of African ancestries are among the most genetically diverse in the world, meaning that a PRS that performs well in one population may not perform well in others [37].

We examined the extent to which the best‐performing PRS captured biological processes related to T2 inflammation, wound healing, and drug response as measured by transcriptomic gene expression modules and clinical biomarkers. Of these three axes of dysregulation, the PRS was mediated the most by T2 inflammation. All three statistically significant quantitative biomarker mediators (blood eosinophil counts, tIgE, and phadiatop sIgE) are classical hallmarks of a T2‐immune response [9]. Prior work has shown correlation between total and specific IgE with potentially nonoverlapping genetic contributions [38]. In our CAAPA data, tIgE, and phadiatop multi‐allergen sIgE are highly correlated (*r* = 0.56, *p* < 2.2e‐16) with similar percent mediation (~38%, *p*.adj < 0.0002), making the deconvolution of their individual contributions difficult. We are also unable to distinguish between pleiotropy (IgE ← genetics→ asthma) and causality (genetics →asthma →IgE or genetics →IgE→asthma). Nonetheless, our statistical mediation demonstrates that a large amount of the relationship between the PRS and asthma phenotype is related to differences in tIgE, and similarly with sIgE.

Also related to T2 inflammation are the two strongest nasal epithelium gene expression modules for the mediation analysis (M2 and M6). The hub gene for module M2, carboxypeptidase A3 (*CPA3*), is a mast cell protease that contributes to the pathology of asthma, including severe disease [39, 40]. Module M6's hub gene was *CEACAM5*, an epithelial gene that is upregulated in severe asthma and is associated with asthma exacerbation; *CEACAM5* is regulated through IL‐13, one of the primary cytokines involved with T2 inflammation [41, 42, 43]. The asthma PRS being mediated primarily through T2 inflammation is not surprising; 80.7% of asthma cases in CAAPA were classified as atopic through elevated tIgE and/or phadiatop sIgE, so it follows that T2‐related biomarkers and gene expression pathways would be mediating the PRS‐asthma relationship. Additionally, transcriptome‐wide association studies have already established that T2‐inflammation‐related gene expression in the nasal epithelium can mediate the relationship between asthma‐associated SNPs and the asthma phenotype [44]. Our results thus support the large body of literature establishing that one of the primary mechanisms captured by asthma genetics is the well‐established T2 inflammatory pathway.

Mediation analysis also pointed to novel transcriptomic networks related to wound healing and asthma medication response. Module M5 explained 11.90% of the asthma‐PRS relationship (*p*.adj = 0.008). Module M5's hub‐gene was fibronectin 1 (*FN1*), which encodes an adhesion protein that has previously been found to be under‐expressed in the airway epithelial tissues of childhood asthma cases; fibronectin can impair wound healing processes in human airway epithelial cells [45]. A second wound healing gene expression module, M23 (hub gene: *ACVR1B*), was also a statistically significant mediator, explaining 11.05% (*p*.adj = 0.015) of the PRS‐asthma relationship. Overall, our results indicate that 11%–12% of the PRS‐asthma relationship may be capturing the effects of impaired wound healing in the nasal epithelium. These findings provide additional credibility in support of our prior findings in CAAPA that the novel wound healing gene expression modules play an important role in the context of asthma and provide additional insights into the limited, but nonzero, extent to which asthma PRSs capture non‐T2 processes.

Mediation by impaired medication response was the weakest out of the three axes of dysregulation. A prior CAAPA study identified associations between modules M4 (hub gene: *NCALD*) and M9 (hub gene: *DNAH5*) with current asthma, with both modules being downregulated in asthma cases. The existing literature points to *NCALD* and *DNAH5* having important roles in the response to asthma medications [19, 46, 47]. We expanded upon that prior work by showing that these two gene expression modules were significant (*p*.adj = 0.049 for both modules) mediators of the PRS‐asthma relationship, mediating 6.8% and 5.7%, respectively. Interestingly, modules M4 and M9 were not directly associated with the asthma PRS in our differential expression analysis. Given the lower mediation assigned to these two modules, we conclude that even though the mediation itself was statistically significant, the extent of this mediation is weak.

Our analysis focused primarily on the African diaspora due to their many asthma health disparities and their underrepresentation in research [14, 15, 16, 17, 18]. A prior investigation of mediation identified that 8% of the PRS‐asthma association was mediated by eosinophils among white British individuals from the UK Biobank, similar to the 7% mediation estimate obtained in CAAPA [6]. Our paper extends these approaches to examine the mediatory effect of the wound healing and medication response through novel axes of transcriptomic dysregulation. A future direction of research would be to conduct similar mediation analyses in other ancestral populations to determine whether the genetic risk signature for asthma captures the wound healing and medication response axes across ancestry groups as noted with the T2 processes.

Asthma is a heterogeneous condition with multiple distinct endotypes. In our CAAPA samples, most asthma cases were atopic (80.7%), and therefore our mediation analysis results implicating a more prominent role of T2 mechanisms may not necessarily be generalizable to a population where nonatopic and T2‐low asthma may be more common. In addition, differences in the overall distributions of both potential mediators and the asthma phenotype between populations limit the generalizability of our mediation results. We may expect to see differences in the overall extent of mediation if this analysis is attempted in other populations with vastly different biomarker distributions or distributions of asthma endotypes.

An underlying, yet untested, assumption of regression‐based mediation analysis is the possibility of reverse causation between the outcome and mediator [48]. This concern is especially relevant as the nasal epithelium samples were collected from prevalent asthma cases, rather than prior to asthma onset. For example, we cannot distinguish between a scenario where genetics predisposes someone to asthma resulting in elevated eosinophils and a scenario where genetics predisposes someone to elevated eosinophils, which then results in asthma. For this reason, we caution against the interpretation of a directional causal relationship, instead interpreting the percent mediation as the extent of the asthma PRS‐asthma relationship that is captured or reflective of a given biological mechanism or biomarker. Additionally, we are unable to fully rule out potential unmeasured confounding from environmental or social determinants of health that could bias our mediation analyses. Finally, even though the limited sample size used in our analysis was sufficient to draw statistically significant conclusions, it is still a limitation of our dataset as other samples of comparable size may have differing distributions of asthma endotypes. Our sample size also precluded extensive subgroup and sub‐phenotype analyses.

Given the lack of family history data within CAAPA, we are unable to contextualize the asthma PRSs in the context of having family history of asthma. Prior work dealing with an asthma PRS (PGS000799, ΔAUC within CAAPA = 0.030) demonstrated that an asthma PRS contributed no additional predictive value over that offered by the combination of family history of asthma and atopy, perinatal factors, and the environment [49]. The caveat to this interpretation is that this PRS was developed using a moderate sample (*n* = 146,984) with overall lower predictive accuracy than the GBMI PRSs. In the context of other complex diseases, prior research has demonstrated that PRSs both recapitulate and provide additional complementary information to familial history, and additional work is needed to evaluate this for asthma [50].

This study provides added value to the existing asthma PRS literature by utilizing individuals from African‐ancestry populations, who have often been underrepresented in genomics research. By utilizing molecular/cellular biomarkers (tIgE, phadiatop sIgE, and eosinophils) and gene expression modules, our study adds to the existing literature by revealing that existing asthma PRSs primarily capture the biology of T2 inflammation, with lesser but still statistically significant contributions from wound healing and medication response processes. The connection through the novel wound healing and medication response axes of dysregulation provides foundational evidence for additional features that may need to be considered in building endotypes for asthma. A future direction of follow‐up would be to take these 3 axes of dysregulation and connect them to asthma subphenotypes.

## Author Contributions

R.N., B.S., K.K., E.E., R.K.J., and R.A.M. performed analysis. M.P.B., S.C., and M.C. were involved in data generation and quality control. W.L., A.P.D., A.A.C., H.W., E.T.N., B.L.G., G.A., O.S., A.G.F., N.N.H., C.O.O., C.N.R., R.C.L., C.A.F., and A.H.L. were involved in patient recruitment. R.A.M., I.R., M.A.T., and G.L.W. contributed to design of data analysis. E.E.K., K.C.B., and R.A.M. were the principal investigators for the study and oversaw the study design. R.N., K.K., G.L.W., and R.A.M. wrote the primary body of the manuscript; all other authors read and contributed to the writing on the manuscript.

## Conflicts of Interest

K.C.B. declares Royalties from UpToDate. The other authors declare no conflicts of interest.

## Supporting information


**Figure S1:** PRS discriminative performance within CAAPA compared to training data sample size. This scatterplot shows the discriminative performance of PRSs within CAAPA, contrasting the change in the area under the ROC curve [ΔAUC = AUC_Expanded Model_—AUC_Base Model_] within CAAPA (*y*‐axis) against the sample size of the PRS's discovery GWAS (*x*‐axis), as previously illustrated in Figure 1. The numbers in the plot correspond to the score's index in Table S3.
**Table S1:** Clinical characteristics of the subset of CAAPA study participants used in the WGCNA module mediation analysis. *p*‐values are presented for the quantitative clinical traits used in the mediation analysis, derived using a 2‐sample *t*‐test. Atopic individuals were defined as those with a tIgE > 100 kU/L and/or Phadiatop sIgE ≥ 0.36 PAU/L. Estimates of genetic similarity were obtained through ADMIXTURE (*k* = 3). Geo. = geometric.
**Table S2:** Overview of PRS match rate between PRS score files and CAAPA imputed genotypes. Score IDs with a * were not calculated due to low match rate (< 75%)
**Table S3:** Characteristics and details about the development of the PRSs applied in this paper, modified from the PGS Catalog metadata. BBJ, Biobank Japan; GABRIEL, A Multidisciplinary Study to Identify the Genetic and Environmental Causes of Asthma in the European Community, GBMI, Global Biobank Meta‐analysis Initative, EUR, European; TAGC, Trans‐National Asthma Genetic Consortium, UKBB, UK Biobank. In this table, ancestry categories were defined based on the PGS Catalog metadata and as described in the papers that derived the PRSs.
**Table S4:** The mediation of polygenic asthma risk through WGCNA gene expression modules. This table summarizes the results of the differentially expressed module analyses, showing the module‐Asthma (Szcesny et al. 2024) and module‐AsthmaPRS associations. In addition, it contains the percent mediation. *p*.adj = Benjamini‐Hochberg corrected *p*‐values. STRING was only run where *p*.adj < 0.05 in any of the three tests.

## Data Availability

The data that support the findings of this study are openly available in GEO at https://www.ncbi.nlm.nih.gov/geo/query/acc.cgi?acc=GSM7701971, reference number GSE240567.

## References

[1] S. F. Thomsen , “Exploring the Origins of Asthma: Lessons From Twin Studies,” European Clinical Respiratory Journal 1, no. Suppl 1 (2014): 5–6, 10.3402/ecrj.v1.25535. PMC4629771 26557247

[2] E. Toskala and D. W. Kennedy , “Asthma Risk Factors,” International Forum of Allergy & Rhinology 5, no. Suppl 1 (2015): 11. 10.1002/alr.21557 PMC7159773 26335830

[3] M. Pividori , N. Schoettler , D. L. Nicolae , C. Ober , and H. K. Im , “Shared and Distinct Genetic Risk Factors for Childhood‐Onset and Adult‐Onset Asthma: Genome‐Wide and Transcriptome‐Wide Studies,” Lancet Respiratory Medicine 7, no. 6 (2019): 509–522. 31036433 10.1016/S2213-2600(19)30055-4 PMC6534440

[4] N. J. Samani , E. Beeston , C. Greengrass , et al., “Polygenic Risk Score Adds to a Clinical Risk Score in the Prediction of Cardiovascular Disease in a Clinical Setting,” European Heart Journal 45, no. 34 (2024): 3152–3160. 38848106 10.1093/eurheartj/ehae342 PMC11379490

[5] T. A. Bond , R. C. Richmond , V. Karhunen , et al., “Exploring the Causal Effect of Maternal Pregnancy Adiposity on Offspring Adiposity: Mendelian Randomisation Using Polygenic Risk Scores,” BMC Medicine 20, no. 1 (2022): 34. 35101027 10.1186/s12916-021-02216-w PMC8805234

[6] M. Dapas , Y. L. Lee , W. Wentworth‐Sheilds , H. K. Im , C. Ober , and N. Schoettler , “Revealing Polygenic Pleiotropy Using Genetic Risk Scores for Asthma,” HGG Advances 4, no. 4 (2023): 100233. 37663543 10.1016/j.xhgg.2023.100233 PMC10474095

[7] M. A. R. Ferreira , R. Mathur , J. M. Vonk , et al., “Genetic Architectures of Childhood‐ and Adult‐Onset Asthma Are Partly Distinct,” American Journal of Human Genetics 104, no. 4 (2019): 665–684. 30929738 10.1016/j.ajhg.2019.02.022 PMC6451732

[8] T. F. Carr and E. Bleecker , “Asthma Heterogeneity and Severity,” World Allergy Organization Journal 9, no. 1 (2016): 41–42. 27980705 10.1186/s40413-016-0131-2 PMC5129643

[9] M. E. Kuruvilla , F. E.‐H. Lee , and G. B. Lee , “Understanding Asthma Phenotypes, Endotypes, and Mechanisms of Disease,” Clinical Reviews in Allergy & Immunology 56, no. 2 (2019): 219–233. 30206782 10.1007/s12016-018-8712-1 PMC6411459

[10] N. Yu , F.‐C. Chen , S. Ota , et al., “Larger Genetic Differences Within Africans Than Between Africans and Eurasians,” Genetics 161, no. 1 (2002): 269–274. 12019240 10.1093/genetics/161.1.269 PMC1462113

[11] S. A. Lambert , L. Gil , S. Jupp , et al., “The Polygenic Score Catalog as an Open Database for Reproducibility and Systematic Evaluation,” Nature Genetics 53, no. 4 (2021): 420–425. 33692568 10.1038/s41588-021-00783-5 PMC11165303

[12] T. Schoeler , D. Speed , E. Porcu , N. Pirastu , J.‐B. Pingault , and Z. Kutalik , “Participation Bias in the UK Biobank Distorts Genetic Associations and Downstream Analyses,” Nature Human Behaviour 7, no. 7 (2023): 1216–1227. 10.1038/s41562-023-01579-9 PMC10365993 37106081

[13] C. Sudlow , J. Gallacher , N. Allen , et al., “UK Biobank: an Open Access Resource for Identifying the Causes of a Wide Range of Complex Diseases of Middle and Old Age,” PLoS Medicine 12, no. 3 (2015): e1001779. 25826379 10.1371/journal.pmed.1001779 PMC4380465

[14] M. F. Perez and M. T. Coutinho , “An Overview of Health Disparities in Asthma,” Yale Journal of Biology and Medicine 94, no. 3 (2021): 497–507. 34602887 PMC8461584

[15] T. Guilbert , R. S. Zeiger , T. Haselkorn , et al., “Racial Disparities in Asthma‐Related Health Outcomes in Children With Severe/Difficult‐to‐Treat Asthma,” Journal of Allergy and Clinical Immunology 7, no. 2 (2019): 568–577. 10.1016/j.jaip.2018.07.050 30172020

[16] B. B. Obeng , F. Hartgers , D. Boakye , and M. Yazdanbakhsh , “Out of Africa: What Can Be Learned From the Studies of Allergic Disorders in Africa and Africans?,” Current Opinion in Allergy and Clinical Immunology 8, no. 5 (2008): 391–397. 18769190 10.1097/ACI.0b013e32830ebb70

[17] N. Pearce , N. Aït‐Khaled , R. Beasley , et al., “Worldwide Trends in the Prevalence of Asthma Symptoms: Phase III of the International Study of Asthma and Allergies in Childhood (ISAAC),” Thorax 62, no. 9 (2007): 758–766. 17504817 10.1136/thx.2006.070169 PMC2117323

[18] C. Vergara , T. Murray , N. Rafaels , et al., “African Ancestry Is a Risk Factor for Asthma and High Total IgE Levels in African Admixed Populations,” Genetic Epidemiology 37, no. 4 (2013): 393–401. 23554133 10.1002/gepi.21702 PMC4051322

[19] B. Szczesny , M. P. Boorgula , S. Chavan , et al., “Multi‐Omics in Nasal Epithelium Reveals Three Axes of Dysregulation for Asthma Risk in the African Diaspora Populations,” Nature Communications 15, no. 1 (2024): 4546. 10.1038/s41467-024-48507-7 PMC11133339 38806494

[20] J. J. Wildfire , P. J. Gergen , C. A. Sorkness , et al., “Development and Validation of the Composite Asthma Severity Index—An Outcome Measure for Use in Children and Adolescents,” Journal of Allergy and Clinical Immunology 129, no. 3 (2012): 694–701. 22244599 10.1016/j.jaci.2011.12.962 PMC3294274

[21] C. Y. Wong , K. W. Yeh , J. L. Huang , et al., “Longitudinal Analysis of Total Serum IgE Levels With Allergen Sensitization and Atopic Diseases in Early Childhood,” Scientific Reports 10, no. 1 (2020): 21278. 33277617 10.1038/s41598-020-78272-8 PMC7718260

[22] A. Poole , C. Urbanek , C. Eng , et al., “Dissecting Childhood Asthma With Nasal Transcriptomics Distinguishes Subphenotypes of Disease,” Journal of Allergy and Clinical Immunology 133, no. 3 (2014): 670–678. 24495433 10.1016/j.jaci.2013.11.025 PMC4043390

[23] Y. H. Tsai , J. S. Parker , I. V. Yang , and S. N. P. Kelada , “Meta‐Analysis of Airway Epithelium Gene Expression in Asthma,” European Respiratory Journal 51, no. 5 (2018): 1701962. 29650561 10.1183/13993003.01962-2017 PMC7395676

[24] S. Das , L. Forer , S. Schönherr , et al., “Next‐Generation Genotype Imputation Service and Methods,” Nature Genetics 48, no. 10 (2016): 1284–1287. 27571263 10.1038/ng.3656 PMC5157836

[25] C. Fuchsberger , G. R. Abecasis , and D. A. Hinds , “minimac2: Faster Genotype Imputation,” Bioinformatics 31, no. 5 (2014): 782–784. 25338720 10.1093/bioinformatics/btu704 PMC4341061

[26] P.‐R. Loh , P. Danecek , P. F. Palamara , et al., “Reference‐Based Phasing Using the Haplotype Reference Consortium Panel,” Nature Genetics 48, no. 11 (2016): 1443–1448. 27694958 10.1038/ng.3679 PMC5096458

[27] D. Taliun , D. N. Harris , M. D. Kessler , et al., “Sequencing of 53,831 Diverse Genomes From the NHLBI TOPMed Program,” Nature 590, no. 7845 (2021): 290–299. 33568819 10.1038/s41586-021-03205-y PMC7875770

[28] S. A. Lambert , B. Wingfield , J. T. Gibson , et al., “The Polygenic Score Catalog: New Functionality and Tools to Enable FAIR Research,” medRxiv. (2024), 2024.05.29.24307783.

[29] H. Wickham , M. Averick , J. Bryan , et al., “Welcome to the Tidyverse,” Journal of Open Source Software 4, no. 43 (2019): 1686.

[30] X. Robin , N. Turck , A. Hainard , et al., “pROC: An Open‐Source Package for R and S+ to Analyze and Compare ROC Curves,” BMC Bioinformatics 12 (2011): 77. 21414208 10.1186/1471-2105-12-77 PMC3068975

[31] M. Nakazawa , “fmsb: Functions for Medical Statistics Book With Some Demographic Data,” (2023).

[32] D. P. MacKinnon , C. M. Lockwood , C. H. Brown , W. Wang , and J. M. Hoffman , “The Intermediate Endpoint Effect in Logistic and Probit Regression,” Clinical Trials 4, no. 5 (2007): 499–513. 17942466 10.1177/1740774507083434 PMC2857773

[33] Y. Wang , S. Namba , E. Lopera , et al., “Global Biobank Analyses Provide Lessons for Developing Polygenic Risk Scores Across Diverse Cohorts,” Cell Genomics 3, no. 1 (2023): 100241. 36777179 10.1016/j.xgen.2022.100241 PMC9903818

[34] D. M. Kothalawala , L. Kadalayil , J. A. Curtin , et al., “Integration of Genomic Risk Scores to Improve the Prediction of Childhood Asthma Diagnosis,” Journal of Personalized Medicine 12, no. 1 (2022): 75, 10.3390/jpm12010075. 35055391 PMC8777841

[35] Y. D. Zhang , A. N. Hurson , H. Zhang , et al., “Assessment of Polygenic Architecture and Risk Prediction Based on Common Variants Across Fourteen Cancers,” Nature Communications 11, no. 1 (2020): 3353. 10.1038/s41467-020-16483-3 PMC7335068 32620889

[36] F. Dudbridge , “Power and Predictive Accuracy of Polygenic Risk Scores,” PLoS Genetics 9, no. 3 (2013): e1003348. 23555274 10.1371/journal.pgen.1003348 PMC3605113

[37] L. Majara , A. Kalungi , N. Koen , et al., “Low and Differential Polygenic Score Generalizability Among African Populations due Largely to Genetic Diversity,” HGG Advances 4, no. 2 (2023): 100184. 36873096 10.1016/j.xhgg.2023.100184 PMC9982687

[38] D. P. Potaczek and M. Kabesch , “Current Concepts of IgE Regulation and Impact of Genetic Determinants,” Clinical and Experimental Allergy 42, no. 6 (2012): 852–871. 22909159 10.1111/j.1365-2222.2011.03953.x

[39] C. Andersson , E. Tufvesson , Z. Diamant , and L. Bjermer , “Revisiting the Role of the Mast Cell in Asthma,” Current Opinion in Pulmonary Medicine 22, no. 1 (2016): 10–17. 26574723 10.1097/MCP.0000000000000228

[40] G. Pejler , “The Emerging Role of Mast Cell Proteases in Asthma,” European Respiratory Journal 54, no. 4 (2019): 1900685. 31371445 10.1183/13993003.00685-2019

[41] U. Hoda , S. Pavlidis , A. T. Bansal , et al., “Clinical and Transcriptomic Features of Persistent Exacerbation‐Prone Severe Asthma in U‐BIOPRED Cohort,” Clinical and Translational Medicine 12, no. 4 (2022): e816. 35474304 10.1002/ctm2.816 PMC9043117

[42] S. Mumby , N. Z. Kermani , J. P. Garnett , et al., “CEACAM5 Is an IL‐13‐Regulated Epithelial Gene That Mediates Transcription in Type‐2 (T2) High Severe Asthma,” Allergy 77, no. 11 (2022): 3463–3466. 35916059 10.1111/all.15465

[43] C. Xu , L. Du , Z. Zeng , F. Chen , Y. Liang , and Y. Guo , “Elevated CEACAM5 Levels in Patients With Asthma,” International Archives of Allergy and Immunology 183, no. 6 (2022): 673–681. 35172310 10.1159/000521754

[44] S. P. Sajuthi , J. L. Everman , N. D. Jackson , et al., “Nasal Airway Transcriptome‐Wide Association Study of Asthma Reveals Genetically Driven Mucus Pathobiology,” Nature Communications 13, no. 1 (2022): 1632. 10.1038/s41467-022-28973-7 PMC8960819 35347136

[45] A. Kicic , T. S. Hallstrand , E. N. Sutanto , et al., “Decreased Fibronectin Production Significantly Contributes to Dysregulated Repair of Asthmatic Epithelium,” American Journal of Respiratory and Critical Care Medicine 181, no. 9 (2010): 889–898. 20110557 10.1164/rccm.200907-1071OC PMC2862303

[46] J. Joo , A. C. Y. Mak , S. Xiao , et al., “Genome‐Wide Association Study in Minority Children With Asthma Implicates DNAH5 in Bronchodilator Responsiveness,” Scientific Reports 12, no. 1 (2022): 12514. 35869121 10.1038/s41598-022-16488-6 PMC9307508

[47] J. E. Sordillo , S. M. Lutz , M. J. McGeachie , et al., “Pharmacogenetic Polygenic Risk Score for Bronchodilator Response in Children and Adolescents With Asthma: Proof‐Of‐Concept,” Journal of Personalized Medicine 11, no. 4 (2021): 319. 33923870 10.3390/jpm11040319 PMC8073919

[48] D. P. MacKinnon , A. J. Fairchild , and M. S. Fritz , “Mediation Analysis,” Annual Review of Psychology 58 (2007): 593–614. 10.1146/annurev.psych.58.110405.085542 PMC2819368 16968208

[49] F. N. Dijk , C. Folkersma , O. Gruzieva , et al., “Genetic Risk Scores Do Not Improve Asthma Prediction in Childhood,” Journal of Allergy and Clinical Immunology 144, no. 3 (2019): 857–860. 31145937 10.1016/j.jaci.2019.05.017

[50] N. Mars , J. V. Lindbohm , P. Della Briotta Parolo , et al., “Systematic Comparison of Family History and Polygenic Risk Across 24 Common Diseases,” American Journal of Human Genetics 109, no. 12 (2022): 2152–2162. 36347255 10.1016/j.ajhg.2022.10.009 PMC9748261

