# Supplementary material for: Mediation of Polygenic Asthma Risk Through Gene Expression
Source: Allergy. 2025 Dec 6;81(4):1089–98. doi: 10.1111/all.70101 (PMC13040662; doi:10.1111/all.70101)
Supplement: Supplementary file 1 — Figure S1: PRS discriminative performance within CAAPA compared to training data sample size. This scatterplot shows the discriminative performance of PRSs within CAAPA, contrasting the change in the area under the ROC curve [ΔAUC = AUCExpanded Model—AUCBase Model] within CAAPA (y‐axis) against the sample size of the PRS's discovery GWAS (x‐axis), as previously illustrated in Figure 1. The numbers in the plot correspond to the score's index in Table S3. Table S1: Clinical characteristics of the subset of CAAPA study participants used in the WGCNA module mediation analysis. p‐values are presented for the quantitative clinical traits used in the mediation analysis, derived using a 2‐sample t‐test. Atopic individuals were defined as those with a tIgE > 100 kU/L and/or Phadiatop sIgE ≥ 0.36 PAU/L. Estimates of genetic similarity were obtained through ADMIXTURE (k = 3). Geo. = geometric. Table S2: Overview of PRS match rate between PRS score files and CAAPA imputed genotypes. Score IDs with a * were not calculated due to low match rate (< 75%) Table S3: Characteristics and details about the development of the PRSs applied in this paper, modified from the PGS Catalog metadata. BBJ, Biobank Japan; GABRIEL, A Multidisciplinary Study to Identify the Genetic and Environmental Causes of Asthma in the European Community, GBMI, Global Biobank Meta‐analysis Initative, EUR, European; TAGC, Trans‐National Asthma Genetic Consortium, UKBB, UK Biobank. In this table, ancestry categories were defined based on the PGS Catalog metadata and as described in the papers that derived the PRSs. Table S4: The mediation of polygenic asthma risk through WGCNA gene expression modules. This table summarizes the results of the differentially expressed module analyses, showing the module‐Asthma (Szcesny et al. 2024) and module‐AsthmaPRS associations. In addition, it contains the percent mediation. p.adj = Benjamini‐Hochberg corrected p‐values. STRING was only run where p.adj < 0.05 in any of the thre [file ALL-81-1089-s001.pdf]

Fig S1. PRS discriminative performance within CAAPA compared to training data sample size. This scatterplot shows the discriminative performance of PRSs within CAAPA, contrasting the change in the area under the ROC curve [$\Delta AUC = AUC_{Expanded\ Model} - AUC_{Base\ Model}$] within CAAPA (y-axis) against the sample size of the PRS's discovery GWAS (x-axis), as previously illustrated in **Fig 1**. The numbers in the plot correspond to the score's index in **Table E3**.

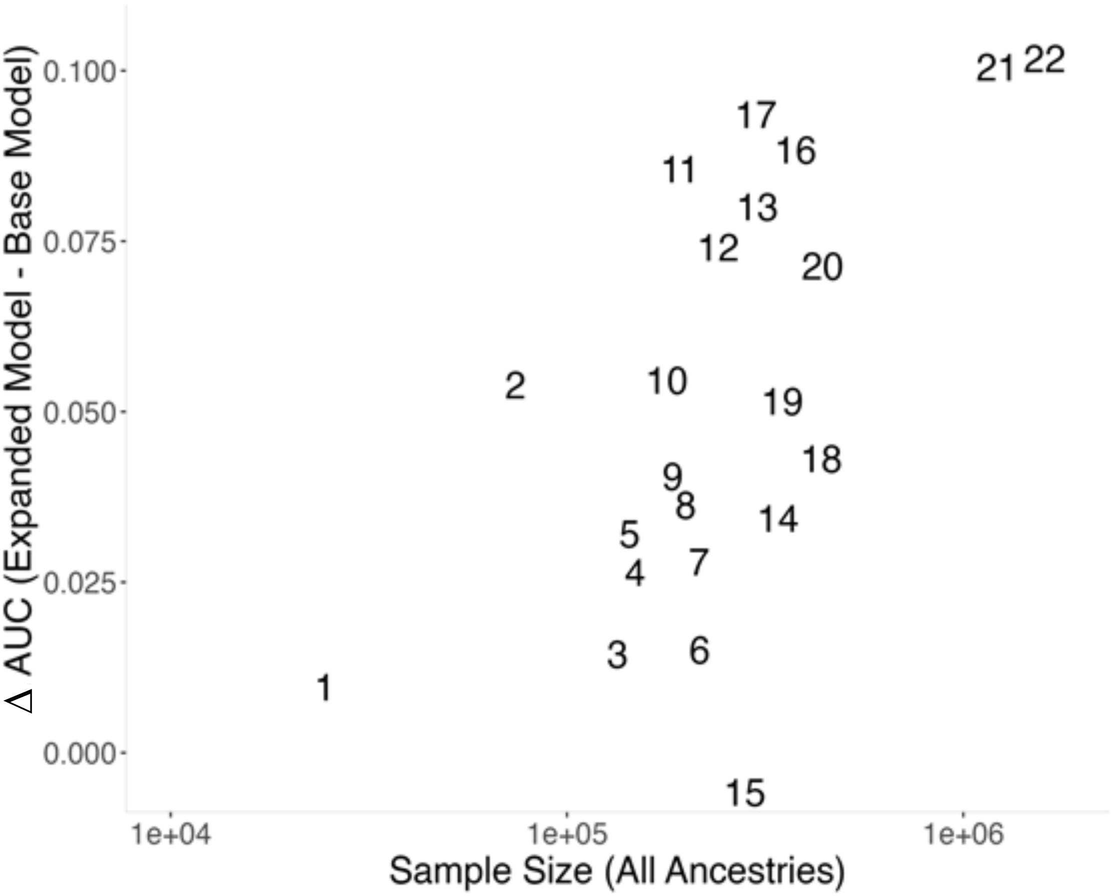

Table S1. Clinical characteristics of the subset of CAAPA study participants used in the WGCNA module mediation analysis. P-values are presented for the quantitative clinical traits used in the mediation analysis, derived using a 2-sample t-test. Atopic individuals were defined as those with a tIgE >100 kU/L and/or Phadiatop sIgE ≥0.36 PAU/L. Estimates of genetic similarity were obtained through ADMIXTURE (k=3). Geo.= geometric.

	Current Asthma Case (N=253)	Never Asthma Control (N=283)	Overall (N=536)	p
**Age (Years)**				
Mean (SD)	31.97 (17.63)	31.80 (16.56)	31.88 (17.06)	
**Sex, N (%)**				
Female	156 (61.7%)	174 (61.5%)	330 (61.6%)	
**Study Site, N(%)**				
Brazil	36 (14.2%)	44 (15.5%)	80 (14.9%)	
Denver	43 (17.0%)	44 (15.5%)	87 (16.2%)	
Chicago	40 (15.8%)	44 (15.5%)	84 (15.7%)	
Baltimore	38 (15.0%)	38 (13.4%)	76 (14.2%)	
Washington DC	19 (7.5%)	43 (15.2%)	62 (11.6%)	
Barbados	44 (17.4%)	37 (13.1%)	81 (15.1%)	
Nigeria	33 (13.0%)	33 (11.7%)	66 (12.3%)	
**Percent similarity to 1kG-YRI**				
Mean (SD)	0.79 (0.19)	0.79 (0.20)	0.79 (0.20)	
**Percent similarity to non-admixed Mao, et al.-AMR samples**				
Mean (SD)	0.02 (0.04)	0.02 (0.04)	0.02 (0.04)	
**Percent similarity to 1kG-CEU**				
Mean (SD)	0.19 (0.17)	0.19 (0.18)	0.19 (0.18)	
**Atopy Status, N(%)**				
Atopic	203 (80.2%)	127 (44.9%)	330 (61.6%)	<0.001
Missing, N (%)	2 (0.8%)	1 (0.4%)	3 (0.6%)	
**Eosinophils (Cells/mm3, offset of**				
Geo. mean (Geo. CV)	157.23 (175.3)	105.77 (159.9)	127.33 (171.2)	<0.001
Missing	12 (4.7%)	9 (3.2%)	21 (3.9%)	
**Neutrophils (Cells/mm3, offset of**				
Geo. mean (Geo. CV)	2748.43 (59.7)	2612.93 (72.6)	2674.37 (66.8)	0.11
Missing, N (%)	19 (7.5%)	8 (2.8%)	27 (5.0%)	
**tIgE (kU/L)**				
Geo. mean (Geo. CV)	107.45 (344.9)	27.23 (300.0)	51.97 (411.8)	<0.001
Missing, N (%)	2 (0.8%)	1 (0.4%)	3 (0.6%)	
**Phadiatop sIgE (PAU/L)**				
Geo. mean (Geo. CV)	3.25 (1646.2)	0.32 (1424.7)	0.95 (2974.8)	<0.001
Missing, N (%)	3 (1.2%)	1 (0.4%)	4 (0.7%)	

Table S2. Overview of PRS match rate between PRS score files and CAAPA imputed genotypes. Score IDs with a * were not calculated due to low match rate (<75%)

Polygenic Score (PGS Catalog ID)	Number of Variants in PRS	Number of Variants Included in CAAPA	Number of Variants Not Included in CAAPA (Unmatched + Failed QC)	Match Rate (%)
PGS000037	15	15	0	100
PGS000799	22	22	0	100
PGS001782	884043	880982	3061	99.7
PGS002061	793375	790817	2558	99.7
PGS002358	920922	918268	2654	99.7
PGS001787	909990	906508	3482	99.6
PGS001849	22679	22597	82	99.6
PGS002677	934369	930813	3556	99.6
PGS002727	985837	982335	3502	99.6
PGS002754	668286	665706	2580	99.6
PGS002311	1109311	1103856	5455	99.5
PGS004178	4924	4459	465	90.6
PGS004176	5210	4692	518	90.1
PGS004177	6770	6089	681	89.9
PGS004179	3963	3552	411	89.6
PGS004180	3641	3255	386	89.4
PGS001346	8508	7421	1087	87.2
PGS001343	6425	5598	827	87.1
PGS001344	6139	5346	793	87.1
PGS001341	6430	5590	840	86.9
PGS004252	212	177	35	83.5
PGS001370	922	760	162	82.4
PGS002248	105	83	22	79
PGS001345	435	336	99	77.2
PGS002579*	503	349	154	69.4
PGS002530*	817	550	267	67.3
PGS002628*	205286	134758	70528	65.6
PGS002383*	3893	2460	1433	63.2
PGS002432*	15843	9204	6639	58.1
PGS002481*	95252	50643	44609	53.2

Table S3. Characteristics and details about the development of the PRSs applied in this paper, modified from the PGS Catalog metadata.

Number in Fig S1	PGS Catalog ID	Phenotype	PGS Method	Continental Ancestry Group	Cohorts	Publication	Sample size [AFR only]
1	PGS000037	Asthma	Pruning and Thresholding	European, Other (unspecified)	GABRIEL	Belsky DW et al. Lancet Respir Med (2013)	0
2	PGS001345	Asthma (diagnosed by doctor)	snpnet	European	UKBB	Tanigawa Y et al. PLoS Genet (2022)	0
3	PGS002358	Asthma	BOLT-LMM	East Asian	BBJ	Weissbrod O et al. Nat Genet (2022)	0
4	PGS002727	Asthma	PRS-CS	African, European, East Asian, Hispanic or Latin American, South Asian	TAGC	Namjou B et al. J Allergy Clin Immunol (2022)	8204
5	PGS000799	Asthma	Genome-wide significant variants	African, European, East Asian, Hispanic or Latin American	TAGC	Dijk FN et al. J Allergy Clin Immunol (2019)	8204
6	PGS004178	Asthma	LASSO	European	UKBB	Raben TG et al. Sci Rep (2023)	0
7	PGS004176	Asthma	LASSO	European	UKBB	Raben TG et al. Sci Rep (2023)	0
8	PGS004179	Asthma	LASSO	European	UKBB	Raben TG et al. Sci Rep (2023)	0
9	PGS004177	Asthma	LASSO	European	UKBB	Raben TG et al. Sci Rep (2023)	0
10	PGS004180	Asthma	LASSO	European	UKBB	Raben TG et al. Sci Rep (2023)	0
11	PGS001344	Asthma (diagnosed by doctor)	snpnet	European	UKBB	Tanigawa Y et al. PLoS Genet (2022)	0
12	PGS001341	Asthma	snpnet	European	UKBB	Tanigawa Y et al. PLoS Genet (2022)	0
13	PGS001343	Asthma (algorithmically-defined)	snpnet	European	UKBB	Tanigawa Y et al. PLoS Genet (2022)	0
14	PGS004252	Asthma	Genome-wide significant SNPs	European	UKBB + TAGC [EUR	Zhu Y et al. Ecotoxicol Environ Saf (2023)	0
15	PGS002248	Childhood asthma	Clumping and Thresholding (PRSice)	European	UKBB	Kothalawala DM et al. J Pers Med (2022)	0
16	PGS002677	Asthma	SBayesR	European	UKBB	Weissbrod O et al. Nat Genet (2022)	0
17	PGS002311	Asthma	BOLT-LMM	European	UKBB	Weissbrod O et al. Nat Genet (2022)	0
18	PGS001849	Asthma	Penalized regression (bigstatsr)	European	UKBB	Prive F et al. Am J Hum Genet (2022)	0
19	PGS002061	Asthma	LDpred2 (bigsnpr)	European	UKBB	Prive F et al. Am J Hum Genet (2022)	0
20	PGS002754	Asthma	PRS-CS	European	UKBB	Mars N et al. Am J Hum Genet (2022)	0
21	PGS001787	Asthma	PRS-CS-auto	African, Native American, East Asian, European, Asian	GBMI	Wang Y et al. Cell Genom (2023)	26083
22	PGS001782	Asthma	PRS-CS-auto	African, Greater Middle Eastern (Middle Eastern, North African or Persian), Native American, East Asian, European, Asian	GBMI	Wang Y et al. Cell Genom (2023)	32658

Abbreviations: OR = Odds Ratio, GABRIEL=A Multidisciplinary Study to Identify the Genetic and Environmental Causes of Asthma in the European Community, UKBB=UK Biobank, BBJ=Biobank Japan, TAGC=Trans-National Asthma Genetic Consortium, GBMI=Global Biobank Meta-analysis Initative, EUR=European.

In this table, ancestry categories were defined based on the PGS Catalog metadata and as described in the papers that derived the PRSs.

Table S3 continued

Number in Fig S1	Sample size [All ancestries]	Incremental Nagelkerke's R-squared	ΔAUC	AUC (Expanded Model)	(90,100) percentile vs. (0,90) percentile		(90,100) percentile vs. (0, 10) percentile		1-SD increase in PRS		Number of Variants in PRS
					Odds Ratio	P-value	Odds Ratio	P-value	OR	P-value	
1	26475	0.007	0.011	0.564	1.24	0.41	1.38	0.44	1.16	0.06	15
2	68835	0.030	0.052	0.605	2.26	2.78E-03	2.99	5.08E-03	1.37	1.07E-04	435
3	124000	0.005	0.013	0.566	1.54	0.1	2.31	3.40E-02	1.13	0.13	920,922
4	146984	0.015	0.029	0.582	1.24	0.42	1.71	0.58	1.41	6.23E-03	985,837
5	146984	0.019	0.030	0.582	1.82	2.51E-02	2.73	8.02E-03	1.28	2.21E-03	22
6	200000	0.004	0.014	0.566	1.05	0.84	1.8	0.14	1.13	0.13	4,924
7	200000	0.012	0.030	0.583	1.38	0.22	2.47	1.85E-02	1.22	1.33E-02	5,210
8	200000	0.016	0.034	0.587	1.18	0.52	2.4	2.38E-02	1.25	5.09E-03	3,963
9	200000	0.021	0.041	0.593	1.16	0.56	1.94	0.07	1.3	1.19E-03	6,770
10	200000	0.034	0.057	0.610	1.52	0.11	3.49	4.27E-03	1.4	4.12E-05	3,641
11	216121	0.061	0.083	0.636	2.05	8.36E-03	5.23	3.02E-05	1.58	4.56E-08	6,139
12	269704	0.055	0.077	0.629	2.28	2.78E-03	6.73	9.34E-06	1.55	1.94E-07	6,430
13	269704	0.059	0.082	0.635	2.23	3.56E-03	8.42	3.10E-06	1.58	8.11E-08	6,425
14	303859	0.018	0.032	0.585	1.48	0.14	3.12	3.54E-03	1.27	2.55E-03	212
15	315541	0.001	-0.003	0.549	1.06	0.83	1.07	0.86	0.95	0.48	105
16	337071	0.057	0.086	0.639	1.89	1.92E-02	3.68	1.63E-03	1.57	1.22E-07	934,369
17	337071	0.071	0.091	0.644	2.29	3.27E-03	4.22	1.56E-03	1.69	4.45E-09	1,109,311
18	391124	0.027	0.041	0.593	1.55	0.1	2.4	2.84E-02	1.35	2.13E-04	22,679
19	391124	0.035	0.054	0.607	1.86	2.10E-02	3.48	1.51E-03	1.4	3.11E-05	793,375
20	394283	0.051	0.069	0.622	2.5	1.11E-03	10.51	3.88E-06	1.53	6.08E-07	668,286
21	1369372	0.092	0.103	0.656	3.75	9.01E-06	13.72	3.11E-07	1.79	4.55E-11	909,990
22	1800785	0.091	0.104	0.657	4.8	6.75E-07	12.46	3.25E-07	1.8	4.18E-11	884,043

Table S4. The mediation of polygenic asthma risk through WGCNA gene expression modules. This table summarizes the results of the differentially expressed module analyses, showing the module-Asthma (Szcesny et al, 2024) and module-AsthmaPRS associations. In addition, it contains the percent mediation. Bolded modules are those with significant mediation.

	Module Description from Szcesny et al 2024					Association with Asthma from Szcesny et al 2024	
Module Number	Most significant DEG	WGCNA hub	STRING hub(s)	# genes in module	# genes with DEG q<0.05	LogFC	p.adj*
**1**	**ETAA1**	**PDCD10**	**EEF1E1**	**88**	**21**	**6.6E-02**	**3.70E-05**
**2**	**PTCHD4**	**CPA3**	**POSTN**	**81**	**45**	**5.5E-01**	**2.40E-14**
3	ENSG00000273599	ENSG00000279476	BCAN, PLAGL1, UGT1A1, PRRT2, GARNL3, CYP2A7	78	15	1.6E-01	1.20E-04
**4**	**SLC13A3**	**IFT172**	**GLRB, NCALD, TP53BP1, PTPRT**	**77**	**17**	**-1.3E-01**	**3.70E-05**
**5**	**FN1**	**DKK3**	**FN1**	**72**	**34**	**-2.8E-01**	**7.60E-09**
**6**	**HS3ST4**	**CEACAM5**	**MET**	**71**	**26**	**3.2E-01**	**9.60E-16**
7	IGKV1-33	IL10RA		68	21	-1.4E-01	0.056
8	VPS18	PRKCSH	ERBB2	**62**	**18**	-5.4E-02	0.006
**9**	**DNAH5**	**DNAH10**	**DNAH5**	**58**	**15**	**-1.3E-01**	**2.50E-04**
10	PARPBP	ASPM	BIRC5, CCNA2	48	11	1.0E-01	0.027
11	FAM169A	ST13	HSP90AB1	48	9	-3.9E-02	0.004
12	VSIG4	HCK		45	18	1.2E-01	0.085
13	BICDL1	GLTP		45	7	7.6E-02	0.056
14	POFUT1	SARS1	VCP	44	11	-5.0E-02	0.003
15	SPTBN1	SF3A1	SMARCA2	44	18	-6.2E-02	0.000
16	CYP2G1P	SLC9C1		38	7	6.2E-02	0.130
17	POR	EDF1		37	11	-2.9E-02	0.170
18	TONSL	PPP1R12C		35	6	4.0E-02	0.120
19	NOTCH2	FAF2		35	8	-2.6E-02	0.110
**20**	**PPP1R9A**	**C16orf89**	**RIMS1, PPP1R9A, NR2F1**	**33**	**14**	**-3.6E-01**	**3.41E-08**
**21**	**GNAS**	**POLD2**	**POLD2, UBB**	**31**	**11**	**-9.2E-02**	**2.20E-04**
22	SAP30	DNAAF6		26	8	7.0E-03	0.790
**23**	**SUSD4**	**ACVR1B**	**CCND1**	**23**	**5**	**-1.6E-01**	**4.60E-08**
24	MT3	RPL7A	RPL23A	21	5	-7.8E-02	0.002

p.adj *=Benjamini-Hochberg corrected p-values
STRING was only run where p.adj<0.05 in any of the three tests

Table S4 continued

Module Number	Association with Asthma PRS LogFC	p.adj**	Percent Mediation: Mediation Estimate	p.adj**
**1**	**0.016**	**0.0860**	**0.063**	**0.0496**
**2**	**0.156**	**3.4E-04**	**0.22**	**< 0.0024**
3	0.041	0.089	0.054	0.064
**4**	**-0.036**	**0.054**	**0.068**	**0.049**
**5**	**-0.072**	**0.015**	**0.119**	**0.008**
**6**	**7.20E-02**	**0.0030**	**0.207**	**0.0024**
7	-0.044	0.30	0.016	0.34
8	-0.016	0.17	0.03	0.15
**9**	**-0.041**	**0.056**	**0.057**	**0.049**
10	0.053	0.05	0.03	0.14
11	-0.01	0.24	0.029	0.19
12	0.039	0.34	0.013	0.45
13	0.018	0.40	0.013	0.44
14	-0.015	0.12	0.038	0.12
15	-0.014	0.100	0.064	0.076
16	-0.015	0.54	-0.008	0.62
17	-0.011	0.37	0.008	0.59
18	-0.002	0.91	-0.001	0.95
19	-0.001	0.91	0.002	0.95
**20**	**-0.089**	**0.023**	**0.109**	**0.018**
**21**	**-0.037**	**0.015**	**0.071**	**0.013**
22	-0.005	0.77	-0.001	0.96
**23**	**-0.042**	**0.015**	**0.111**	**0.015**
24	-0.013	0.37	0.025	0.38
